# The genome sequence of *Philonthus cognatus* (Stephens, 1832) (Coleoptera, Staphylinidae), a rove beetle

**DOI:** 10.12688/wellcomeopenres.19336.1

**Published:** 2023-04-13

**Authors:** Liam M Crowley, Mark Telfer, Michael Geiser, University of Oxford and Wytham Woods Genome Acquisition Lab, John F. Mulley, Natural History Museum Genome Acquisition Lab, Darwin Tree of Life Barcoding collective, Wellcome Sanger Institute Tree of Life programme, Wellcome Sanger Institute Scientific Operations: DNA Pipelines collective, Tree of Life Core Informatics collective, Darwin Tree of Life Consortium

**Affiliations:** 1 University of Oxford, Oxford, England, UK; 2 independent Researcher, Wytham, England, UK; 3 Natural History Museum, London, England, UK; 4 School of Natural Sciences, Bangor University, Bangor, Wales, UK

**Keywords:** Philonthus cognatus, rove beetle, genome sequence, chromosomal, Coleoptera

## Abstract

We present a genome assembly from an individual male *Philonthus cognatus* (a rove beetle; Arthropoda; Insecta; Coleoptera; Staphylinidae). The genome sequence is 1,030.6 megabases in span. Most of the assembly is scaffolded into 12 chromosomal pseudomolecules, including the X and Y sex chromosomes. The mitochondrial genome has also been assembled and is 20.7 kilobases in length. Gene annotation of this assembly on Ensembl identified 29,629 protein coding genes.

## Species taxonomy

Eukaryota; Metazoa; Ecdysozoa; Arthropoda; Hexapoda; Insecta; Pterygota; Neoptera; Endopterygota; Coleoptera; Polyphaga; Staphyliniformia; Staphylinidae; Staphylininae group; Staphylininae; Staphylinini; *Philonthus, Philonthus cognatus* (Stephens, 1832) (NCBI:txid346820).

## Background

*Philonthus cognatus* is a relatively large (8–11 mm), widespread rove beetle (family Staphylinidae), commonly found in damp soils in woodland and grassland habitats across the UK and western Palearctic. It was introduced into North America in the 19th century and is currently present in the USA and Canada ( Majka & Klimaszewski, 2008). Adults are typically black in colour, although some individuals may have a bronze or greenish sheen, and the underside of the basal antennal segment is yellow, making members of this species one of the more easily recognised UK rove beetles. Breeding takes place in late spring to early summer and as a result there are two peaks in abundance, with adults most commonly found in the spring and early summer (March to June) and again in autumn (August to October) ( NBN Atlas Partnership, 2021), although they are present all year round. *P. cognatus,* like many other rove beetles, are important predators of agricultural pests such as aphids ( Dennis & Wratten, 1991; Kollat-Palenga & Basedow, 2000).

The species was named by James Stephens in his multi-volume synopsis of British insects, in the 1832 volume perhaps more famous for referencing some specimens collected by a “C. Darwin Esq.” while he was still a student at Cambridge ( Barlow, 1958; Stephens, 1832). Stephens described *P. cognatus* as being found “within the metropolitan district, but not common”, which seems to contradict their current status as a very common and widespread beetle across the UK and their ‘least concern’ classification in a recent review of the status of the beetles of Great Britain ( Boyce, 2022).

Rove beetles possess defensive glands that produce complex chemical secretions for defence ( Brückner *et al.*, 2021; Parker, 2017) or that have antimicrobial activity ( Lusebrink *et al.*, 2008), and the *P. cognatus* genome assembly will likely prove useful in identifying the biosynthetic pathways involved in the production of these secretions, as well as resolving the polyphyletic status of the species-rich genus *Philonthus* ( Chani-Posse *et al.*, 2018).

### Genome sequence report

**Figure 1.**
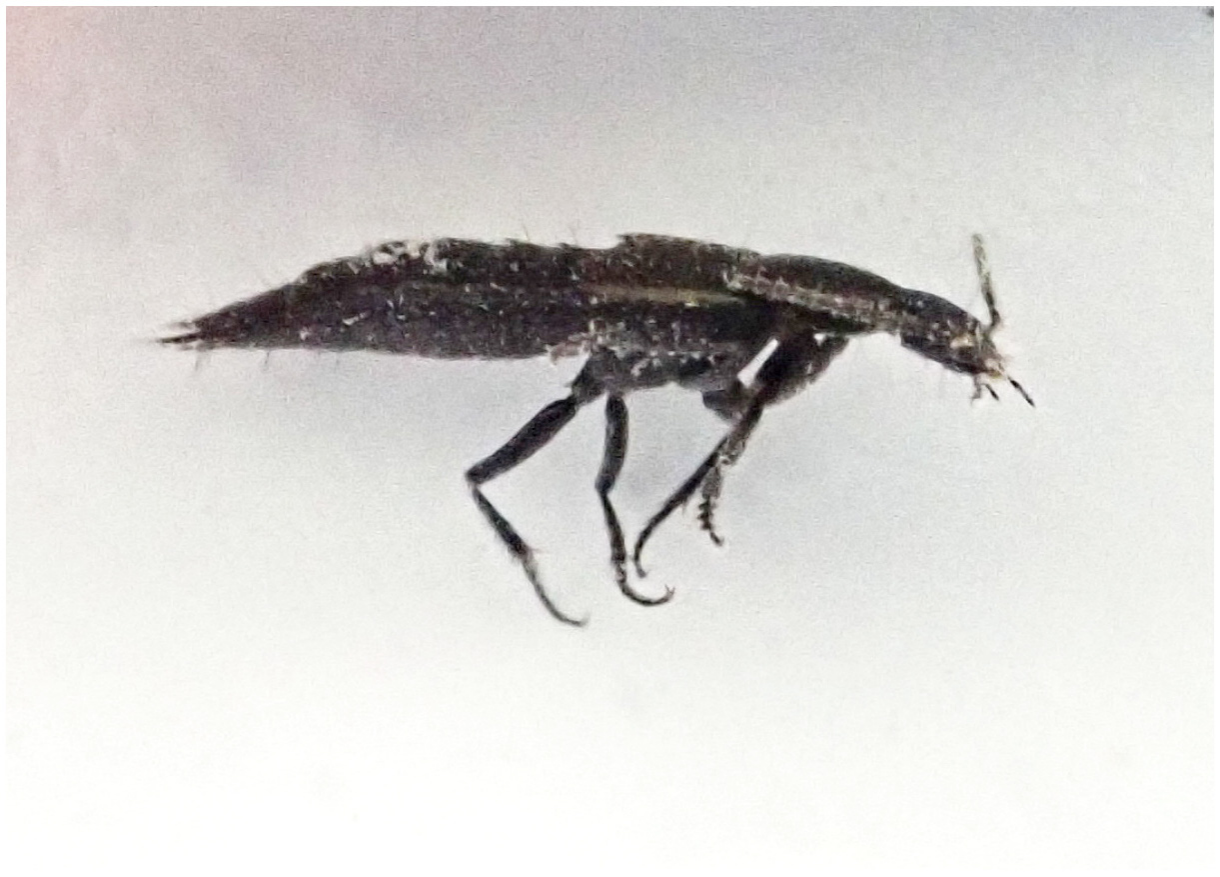
Photograph of the *Philonthus cognatus* (icPhiCogn1) specimen used for genome sequencing.

The genome was sequenced from one male *Philonthus cognatus* ( Figure 1) collected from Wytham Woods, Oxfordshire, UK (latitude 51.77, longitude –1.34). A total of 29-fold coverage in Pacific Biosciences single-molecule HiFi long reads and 47-fold coverage in 10X Genomics read clouds were generated. Primary assembly contigs were scaffolded with chromosome conformation Hi-C data. Manual assembly curation corrected four missing or mis-joins and removed two haplotypic duplications, reducing the assembly length by 0.31% and the scaffold number by 2.17%, and increasing the scaffold N50 by 0.67%.

**Figure 2.**
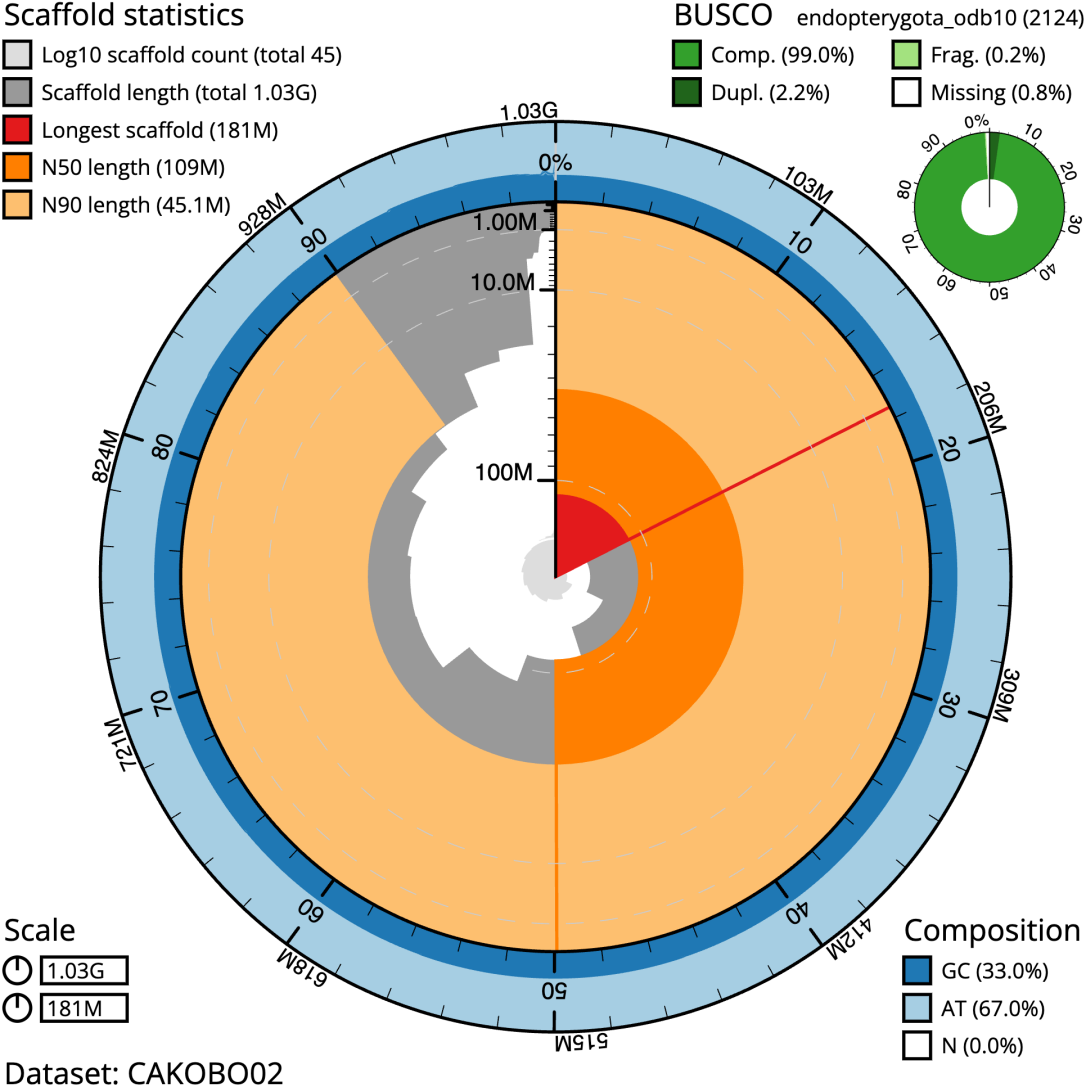
Genome assembly of *Philonthus cognatus*, icPhiCogn1.2: metrics. The BlobToolKit Snailplot shows N50 metrics and BUSCO gene completeness. The main plot is divided into 1,000 size-ordered bins around the circumference with each bin representing 0.1% of the 1,030,568,945 bp assembly. The distribution of scaffold lengths is shown in dark grey with the plot radius scaled to the longest scaffold present in the assembly (181,054,249 bp, shown in red). Orange and pale-orange arcs show the N50 and N90 scaffold lengths (108,863,099 and 45,099,272 bp), respectively. The pale grey spiral shows the cumulative scaffold count on a log scale with white scale lines showing successive orders of magnitude. The blue and pale-blue area around the outside of the plot shows the distribution of GC, AT and N percentages in the same bins as the inner plot. A summary of complete, fragmented, duplicated and missing BUSCO genes in the endopterygota_odb10 set is shown in the top right. An interactive version of this figure is available at https://blobtoolkit.genomehubs.org/view/icPhiCogn1.2/dataset/CAKOBO02/snail.

The final assembly has a total length of 1,030.6 Mb in 45 sequence scaffolds with a scaffold N50 of 10.8.9 Mb ( Table 1). Most (98.54%) of the assembly sequence was assigned to 12 chromosomal-level scaffolds, representing 10 autosomes, and the X and Y sex chromosomes. Chromosome-scale scaffolds confirmed by the Hi-C data are named in order of size ( Figure 2– Figure 5; Table 2). While not fully phased, the assembly deposited is of one haplotype. Contigs corresponding to the second haplotype have also been deposited. The mitochondrial genome was also assembled and can be found as a contig within the multifasta file of the genome submission.

**Table 1.** Genome data for *Philonthus cognatus*, icPhiCogn1.2.

Project accession data		
Assembly identifier	icPhiCogn1.2	
Species	*Philonthus cognatus*	
Specimen	icPhiCogn1	
NCBI taxonomy ID	346820	
BioProject	PRJEB50787	
BioSample ID	SAMEA8603235	
Isolate information	icPhiCogn1.2, abdomen (genome sequencing), head (Hi-C scaffolding) icPhiCogn2, thorax (RNA sequencing)	
Assembly metrics *		*Benchmark*
Consensus quality (QV)	55.8	*≥ 50*
*k*-mer completeness	99.99%	*≥ 95%*
BUSCO **	C:99.0%[S:96.8%,D:2.2%], F:0.2%,M:0.8%,n:2,124	*C ≥ 95%*
Percentage of assembly mapped to chromosomes	98.54%	*≥ 95%*
Sex chromosomes	X and Y	*localised homologous pairs*
Organelles	Mitochondrial genome assembled	*complete single alleles*
Raw data accessions		
PacificBiosciences SEQUEL II	ERR8575390, ERR8575391	
10X Genomics Illumina	ERR8571676–ERR8571679	
Hi-C Illumina	ERR8571680	
PolyA RNA-Seq Illumina	ERR10378003	
Genome assembly		
Assembly accession	GCA_932526585.2	
*Accession of alternate haplotype*	GCA_932526485.1	
Span (Mb)	1,030.6	
Number of contigs	131	
Contig N50 length (Mb)	29.5	
Number of scaffolds	45	
Scaffold N50 length (Mb)	108.9	
Longest scaffold (Mb)	181.1	
Genome annotation		
Number of protein-coding genes	29,629	
Number of gene transcripts	29,922	

* Assembly metric benchmarks are adapted from column VGP-2020 of “Table 1: Proposed standards and metrics for defining genome assembly quality” from ( Rhie *et al.*, 2021). ** BUSCO scores based on the endopterygota_odb10 BUSCO set using v5.3.2. C = complete [S = single copy, D = duplicated], F = fragmented, M = missing, n = number of orthologues in comparison. A full set of BUSCO scores is available at https://blobtoolkit.genomehubs.org/view/icPhiCogn1.2/dataset/CAKOBO02/busco.

**Figure 3.**
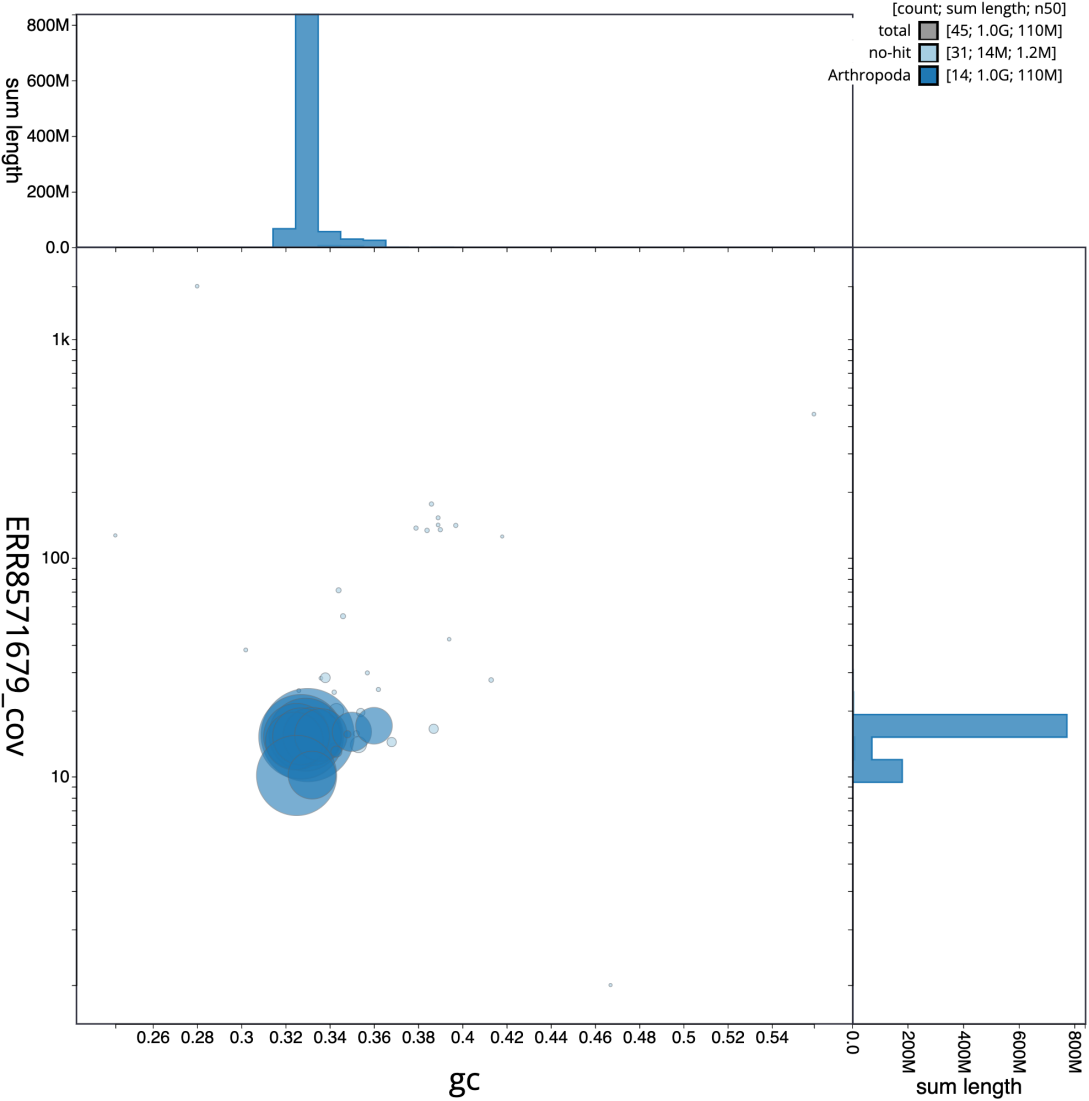
Genome assembly of *Philonthus cognatus*, icPhiCogn1.2: GC coverage. BlobToolKit GC-coverage plot. Scaffolds are coloured by phylum. Circles are sized in proportion to scaffold length. Histograms show the distribution of scaffold length sum along each axis. An interactive version of this figure is available at https://blobtoolkit.genomehubs.org/view/icPhiCogn1.2/dataset/CAKOBO02/blob.

**Figure 4.**
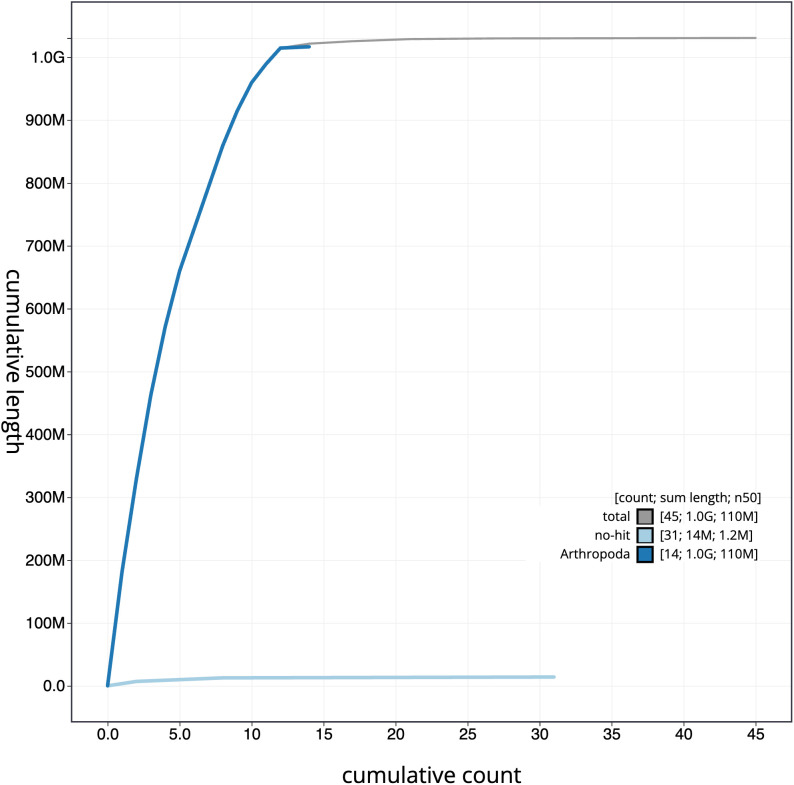
Genome assembly of *Philonthus cognatus*, icPhiCogn1.2: cumulative sequence. BlobToolKit cumulative sequence plot. The grey line shows cumulative length for all scaffolds. Coloured lines show cumulative lengths of scaffolds assigned to each phylum using the buscogenes taxrule. An interactive version of this figure is available at https://blobtoolkit.genomehubs.org/view/icPhiCogn1.2/dataset/CAKOBO02/cumulative.

**Figure 5.**
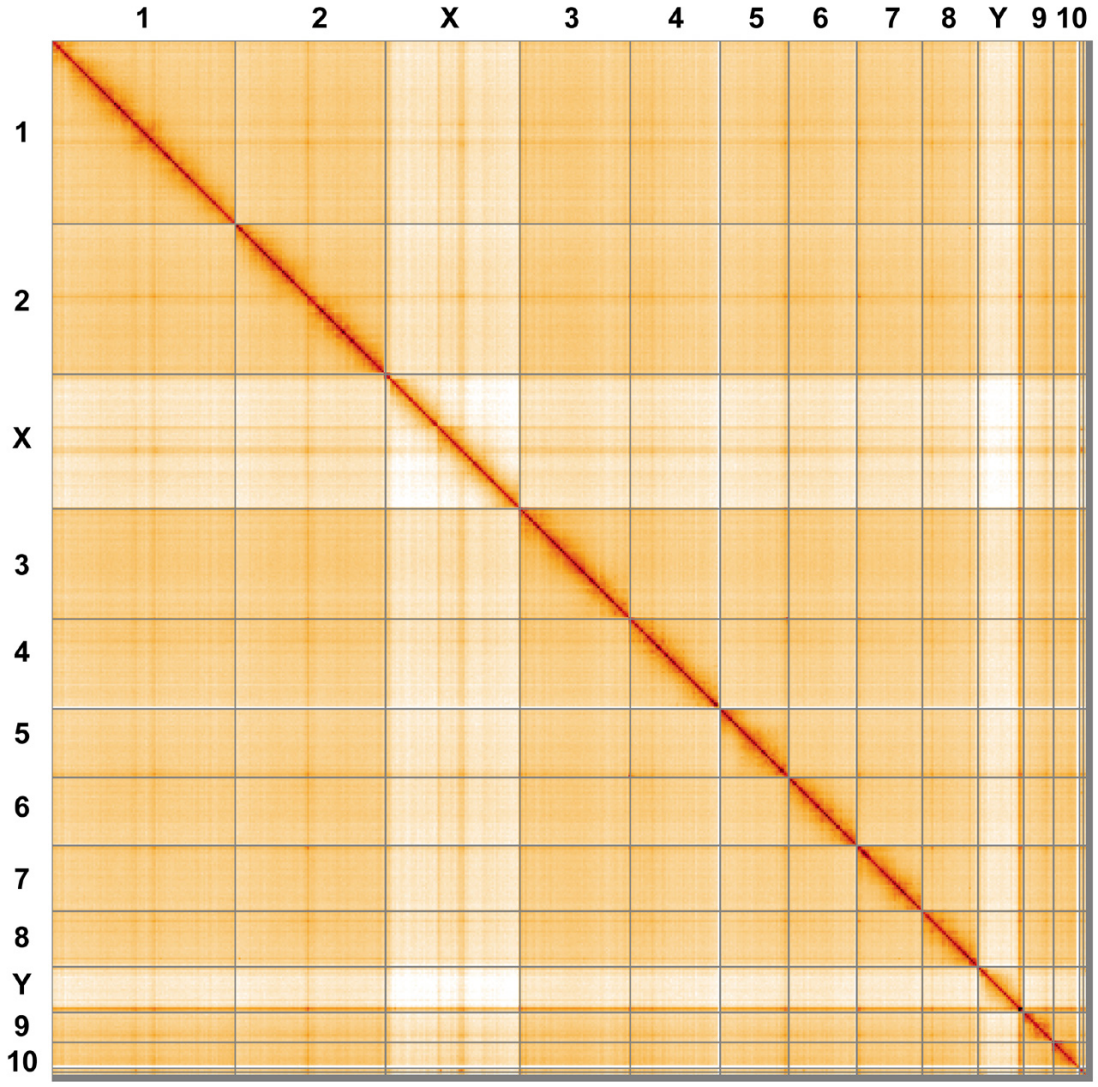
Genome assembly of *Philonthus cognatus*, icPhiCogn1.2: Hi-C contact map. Hi-C contact map of the icPhiCogn1.2 assembly, visualised using HiGlass. Chromosomes are shown in order of size from left to right and top to bottom. An interactive version of this figure may be viewed at https://genome-note-higlass.tol.sanger.ac.uk/l/?d=L6nEPxeIQ1KDr06GZSRUKQ.

**Table 2.** Chromosomal pseudomolecules in the genome assembly of *Philonthus cognatus*, icPhiCogn1.

INSDC accession	Chromosome	Size (Mb)	GC%
OW052241.1	1	181.05	33
OW052242.1	2	148.37	32.7
OW052244.1	3	108.86	32.9
OW052245.1	4	88.74	32.5
OW052246.1	5	67.67	33.2
OW052247.1	6	67.18	32.4
OW052248.1	7	64.79	32.7
OW052249.1	8	55.01	33.6
OW052251.1	9	29.49	35
OW052252.1	10	25.51	36
OW052243.1	X	132.81	32.5
OW052250.1	Y	45.1	33.2
OW052253.2	MT	0.02	28.1
-	unplaced	15.97	35.2

The estimated Quality Value (QV) of the final assembly is 55.8 with *k*-mer completeness of 99.99%, and the assembly has a BUSCO v5.3.2 ( Manni *et al.*, 2021) completeness of 99.0% (single = 96.8%, duplicated = 2.2%), using the endopterygota_odb10 reference set ( *n* = 2,124).

### Genome annotation report

The *Philonthus cognatus* genome assembly GCA_932526585.1 was annotated using the Ensembl rapid annotation pipeline ( Table 1; Ensembl accession number GCA_932526585.1). The resulting annotation includes 29,922 transcribed mRNAs from 29,629 protein-coding genes.

## Methods

### Sample acquisition and nucleic acid extraction

A male *P. cognatus* (icPhiCogn1) was collected from Wytham Woods, Oxfordshire (biological vice-county: Berkshire), UK (latitude 51.77, longitude –1.34) on 8 December 2020. The specimen was taken from woodland habitat by Liam Crowley (University of Oxford) by potting. The specimen was identified by Mark Telfer (independent researcher) and snap-frozen on dry ice.

A second *P. cognatus* specimen (icPhiCogn2) was collected from the Wildlife Garden at the Natural History Museum, London, UK (latitude 51.5, longitude –0.18) on 1 July 2021. The specimen was picked by hand by Michael Geiser (Natural History Museum). The specimen was identified by the collector and preserved at –80°C. The specimen icPhiCogn2 was used for RNA sequencing.

DNA was extracted at the Tree of Life laboratory, Wellcome Sanger Institute (WSI). The icPhiCogn1 sample was weighed and dissected on dry ice with head tissue set aside for Hi-C sequencing. Abdomen tissue was disrupted using a Nippi Powermasher fitted with a BioMasher pestle. High molecular weight (HMW) DNA was extracted using the Qiagen MagAttract HMW DNA extraction kit. Low molecular weight DNA was removed from a 20-ng aliquot of extracted DNA using the 0.8X AMpure XP purification kit prior to 10X Chromium sequencing; a minimum of 50 ng DNA was submitted for 10X sequencing. HMW DNA was sheared into an average fragment size of 12–20 kb in a Megaruptor 3 system with speed setting 30. Sheared DNA was purified by solid-phase reversible immobilisation using AMPure PB beads with a 1.8X ratio of beads to sample to remove the shorter fragments and concentrate the DNA sample. The concentration of the sheared and purified DNA was assessed using a Nanodrop spectrophotometer and Qubit Fluorometer and Qubit dsDNA High Sensitivity Assay kit. Fragment size distribution was evaluated by running the sample on the FemtoPulse system.

RNA was extracted from thorax tissue of icPhiCogn2 in the Tree of Life Laboratory at the WSI using TRIzol, according to the manufacturer’s instructions. RNA was then eluted in 50 μL RNAse-free water and its concentration assessed using a Nanodrop spectrophotometer and Qubit Fluorometer using the Qubit RNA Broad-Range (BR) Assay kit. Analysis of the integrity of the RNA was done using Agilent RNA 6000 Pico Kit and Eukaryotic Total RNA assay.

### Sequencing

Pacific Biosciences HiFi circular consensus and 10X Genomics read cloud DNA sequencing libraries were constructed according to the manufacturers’ instructions. Poly(A) RNA-Seq libraries were constructed using the NEB Ultra II RNA Library Prep kit. DNA and RNA sequencing was performed by the Scientific Operations core at the WSI on Pacific Biosciences SEQUEL II (HiFi), Illumina NovaSeq 6000 (RNA-Seq and 10X) instruments. Hi-C data were also generated from head tissue of icPhiCogn1 using the Arima v2 kit and sequenced on the Illumina NovaSeq 6000 instrument.

### Genome assembly, curation and evaluation

Assembly was carried out with Hifiasm ( Cheng *et al.*, 2021) and haplotypic duplication was identified and removed with purge_dups ( Guan *et al.*, 2020). One round of polishing was performed by aligning 10X Genomics read data to the assembly with Long Ranger ALIGN, calling variants with FreeBayes ( Garrison & Marth, 2012). The assembly was then scaffolded with Hi-C data ( Rao *et al.*, 2014) using YaHS ( Zhou *et al.*, 2023). The assembly was checked for contamination and corrected using the gEVAL system ( Chow *et al.*, 2016) as described previously ( Howe *et al.*, 2021). Manual curation was performed using gEVAL, HiGlass ( Kerpedjiev *et al.*, 2018) and Pretext ( Harry, 2022). The mitochondrial genome was assembled using MitoHiFi ( Uliano-Silva *et al.*, 2022), which performed annotation using MitoFinder ( Allio *et al.*, 2020).

To evaluate the assembly, MerquryFK was used to estimate consensus quality (QV) scores and *k*-mer completeness ( Rhie *et al.*, 2020). The genome was analysed and BUSCO scores ( Manni *et al.*, 2021; Simão *et al.*, 2015) were generated within the BlobToolKit environment ( Challis *et al.*, 2020). Table 3 contains a list of software tool versions and sources.

**Table 3.** Software tools: versions and sources.

Software tool	Version	Source
BlobToolKit	4.0.7	https://github.com/blobtoolkit/blobtoolkit
BUSCO	5.3.2	https://gitlab.com/ezlab/busco
FreeBayes	1.3.1-17-gaa2ace8	https://github.com/freebayes/freebayes
gEVAL	N/A	https://geval.org.uk/
Hifiasm	0.12	https://github.com/chhylp123/hifiasm
HiGlass	1.11.6	https://github.com/higlass/higlass
Long Ranger ALIGN	2.2.2	https://support.10xgenomics.com/genome-exome/software/pipelines/latest/advanced/other-pipelines
Merqury	MerquryFK	https://github.com/thegenemyers/MERQURY.FK
MitoHiFi	2	https://github.com/marcelauliano/MitoHiFi
PretextView	0.2	https://github.com/wtsi-hpag/PretextView
purge_dups	1.2.3	https://github.com/dfguan/purge_dups
YaHS	1	https://github.com/c-zhou/yahs

### Genome annotation

The BRAKER2 pipeline ( Brůna *et al.*, 2021) was used in the default protein mode to generate annotation for the *Philonthus cognatus* assembly (GCA_932526585.1) in Ensembl Rapid Release.

### Ethics and compliance issues

The materials that have contributed to this genome note have been supplied by a Darwin Tree of Life Partner. The submission of materials by a Darwin Tree of Life Partner is subject to the Darwin Tree of Life Project Sampling Code of Practice. By agreeing with and signing up to the Sampling Code of Practice, the Darwin Tree of Life Partner agrees they will meet the legal and ethical requirements and standards set out within this document in respect of all samples acquired for, and supplied to, the Darwin Tree of Life Project. All efforts are undertaken to minimise the suffering of animals used for sequencing. Each transfer of samples is further undertaken according to a Research Collaboration Agreement or Material Transfer Agreement entered into by the Darwin Tree of Life Partner, Genome Research Limited (operating as the Wellcome Sanger Institute), and in some circumstances other Darwin Tree of Life collaborators.

## Data Availability

European Nucleotide Archive: *Philonthus cognatus* (rove beetle). Accession number PRJEB50787; https://identifiers.org/ena.embl/PRJEB50787 ( Wellcome Sanger Institute, 2022) The genome sequence is released openly for reuse. The *Philonthus cognatus* genome sequencing initiative is part of the Darwin Tree of Life (DToL) project. All raw sequence data and the assembly have been deposited in INSDC databases. Raw data and assembly accession identifiers are reported in Table 1.

## Author information

Members of the University of Oxford and Wytham Woods Genome Acquisition Lab are listed here: https://doi.org/10.5281/zenodo.4789928.

Members of the Darwin Tree of Life Barcoding collective are listed here: https://doi.org/10.5281/zenodo.4893703.

Members of the Wellcome Sanger Institute Tree of Life programme are listed here: https://doi.org/10.5281/zenodo.4783585.

Members of Wellcome Sanger Institute Scientific Operations: DNA Pipelines collective are listed here: https://doi.org/10.5281/zenodo.4790455.

Members of the Tree of Life Core Informatics collective are listed here: https://doi.org/10.5281/zenodo.5013541.

Members of the Darwin Tree of Life Consortium are listed here: https://doi.org/10.5281/zenodo.4783558.
