# Supplementary material for: The genome sequence of Philonthus cognatus (Stephens, 1832) (Coleoptera, Staphylinidae), a rove beetle
Source: Wellcome Open Res. 2023 Apr 13;8:169. [Version 1] doi: 10.12688/wellcomeopenres.19336.1 (PMC10333781; doi:10.12688/wellcomeopenres.19336.1)
Supplement: Peer review history

# Peer review: Reviewer response for version 1

**DOI:** 10.21956/wellcomeopenres.21421.r61973

**Published:** 2023-07-10

**Authors:** Ladislav Bocak

**Affiliations:** 1 Biodiversity & Molecular Evolution, Czech Advanced Technology and Research Institute, Olomouc, Czech Republic

Crowley et al. have presented a chromosome-level genome assembly of an individual male Philontus cognatus, a rove beetle from the Staphylinidae family in Coleoptera: Polyphaga. The genome sequence data has been scaffolded into 12 chromosomal pseudomolecules, with the inclusion of the assembled X and Y sex chromosome. The total genome size is over 1 GB, and the assembly is of highly complete. The study is a great contribution to the sources for further phylogenomic research.

 The authors include a photograph of the sequenced specimen, but in contrast with the quality of the assembly, the photo is extremely poor and does not contribute to the presentation (lateral view with low resolution and poor focus). I would recommend that the authors provide a more detailed higher classification of the genus – subfamily, superfamily, and suborder.  

 The presentation of the research is clear and holds importance for future advancements in the field.

Are sufficient details of methods and materials provided to allow replication by others?

Yes

Is the rationale for creating the dataset(s) clearly described?

Yes

Are the datasets clearly presented in a useable and accessible format?

Yes

Are the protocols appropriate and is the work technically sound?

Yes

Reviewer Expertise:

entomology, phylogenomics

I confirm that I have read this submission and believe that I have an appropriate level of expertise to confirm that it is of an acceptable scientific standard.

# Peer review: Reviewer response for version 1

**DOI:** 10.21956/wellcomeopenres.21421.r61969

**Published:** 2023-07-10

**Authors:** Danilo T Amaral

**Affiliations:** 1 Universidade Federal de Sao Carlos, São Carlos, State of São Paulo, Brazil

I had the pleasure of reviewing the publication by Crowley et al., titled "The genome sequence of Philonthus cognatus (Stephens, 1832) (Coleoptera, Staphylinidae), a rove beetle." This study presents the second chromosome-level genome assembly for a member of the Staphylinidae family, offering valuable insights into the genome structure of this group. The high-quality assembly of genomes, particularly at the chromosome level, is pivotal in advancing scientific research. Accurately deciphering the complete genetic blueprint of an organism enables researchers to gain a comprehensive understanding of its structure, function, and evolutionary history. The manuscript provided well-written and detailed methodologies and results. As a reader, I have a minor suggestion. While the figures and tables effectively demonstrate the assembly quality, it may be beneficial to include Figure 4 as supplementary material, based on the sequencing and assembly methodologies. Additionally, it would be interesting to know if any previous studies have performed the karyotyping of this species (or closely related species) to determine the number of chromosomes. Including this information in the text would further enhance the study's context and significance.

Are sufficient details of methods and materials provided to allow replication by others?

Yes

Is the rationale for creating the dataset(s) clearly described?

Yes

Are the datasets clearly presented in a useable and accessible format?

Yes

Are the protocols appropriate and is the work technically sound?

Yes

Reviewer Expertise:

Bioinformatics

I confirm that I have read this submission and believe that I have an appropriate level of expertise to confirm that it is of an acceptable scientific standard.
